# Respective contribution of intensive care unit-acquired limb muscle and severe diaphragm weakness on weaning outcome and mortality: a post hoc analysis of two cohorts

**DOI:** 10.1186/s13054-019-2650-z

**Published:** 2019-11-21

**Authors:** Martin Dres, Boris Jung, Nicolas Molinari, Federico Manna, Bruno-Pierre Dubé, Gerald Chanques, Thomas Similowski, Samir Jaber, Alexandre Demoule

**Affiliations:** 1 AP-HP, Sorbonne Université, Hôpital Pitié-Salpêtrière, Service de Pneumologie, Médecine intensive – Réanimation (Département “R3S”), F-75013 Paris, France; 2 AP-HP, Groupe Hospitalier Pitié-Salpêtrière Charles Foix, Intensive Care Unit and Respiratory Division (Département “R3S”), F-75013 Paris, France; 3 Montpellier School of Medicine, University of Montpellier, INSERM U1046, CNRS UMR 9214, Montpellier, France; 4 Medical Intensive Care Unit, Lapeyronie University Hospital, Montpellier, France; 5 Department of Statistics, CHU Montpellier, IMAG, CNRS, Univ Montpellier, Montpellier, France; 6 Intensive Care and Anesthesiology Department, Saint Eloi Hospital, Montpellier, France; 7 Service de Pneumologie, Médecine Intensive et Réanimation, Groupe Hospitalier Pitié-Salpêtrière, 47-83 Boulevard de l’Hôpital, 75651 Paris Cedex 13, France

**Keywords:** Diaphragm, Muscles, Weakness, Magnetic stimulation, Intensive care unit, Mechanical ventilation, Weaning

## Abstract

**Background:**

Intensive care unit (ICU)-acquired weakness (ICU-AW) and ICU-acquired diaphragm dysfunction (ICU-DD) occur frequently in mechanically ventilated (MV) patients. It is unknown whether they have different risk factors and different impacts on outcome. This study was designed to (1) describe the respective risk factors associated with ICU-AW and severe ICU-DD and (2) evaluate the respective impact of ICU-AW and severe ICU-DD on outcome.

**Methods:**

Post hoc analysis of two prospective cohort studies conducted in two ICUs. In patients mechanically ventilated for at least 24 h undergoing a first spontaneous breathing trial, severe ICU-DD was defined as diaphragm twitch pressure < 7 cmH_2_O and ICU-AW was defined as Medical Research Council Score < 48.

**Results:**

One hundred sixteen patients were assessed. Factors independently associated with severe ICU-DD were age, longer duration of MV, and exposure to sufentanil, and those factors associated with ICU-AW were longer duration of MV and exposure to norepinephrine. Severe ICU-DD (OR 3.56, *p* = 0.008), but not ICU-AW, was independently associated with weaning failure (59%). ICU-AW (OR 4.30, *p* = 0.033), but not severe ICU-DD, was associated with ICU mortality. Weaning failure and mortality rate were higher in patients with both severe ICU-DD and ICU-AW (86% and 39%, respectively) than in patients with either severe ICU-DD (64% and 0%) or ICU-AW (63% and 13%).

**Conclusion:**

Severe ICU-DD and ICU-AW have different risk factors and different impacts on weaning failure and mortality. The impact of the combination of ICU-DD and ICU-AW is more pronounced than their individual impact.

## Background

Intensive care unit (ICU)-acquired weakness (ICU-AW) and ICU-acquired diaphragm dysfunction (ICU-DD) are two well-described complications observed in critically ill patients undergoing mechanical ventilation (MV) [1–7]. ICU-AW and ICU-DD are associated with difficult and prolonged weaning and poorer outcomes [5, 8, 9]. Although ICU-AW and ICU-DD share similar characteristics, their coexistence does not seem to be strongly correlated [5, 9], suggesting that they may be associated with different risk factors [5].

Several studies have evaluated the interactions between respiratory and limb muscle dysfunction in critically ill patients [5, 8, 9], but few of them have assessed diaphragm strength by twitch tracheal pressure in response to bilateral phrenic nerve stimulation (Ptr,stim), which is recognized to be the gold standard [5, 8–10]. In addition, these studies present a number of limitations. Firstly, they included a limited number of patients, making it difficult to evaluate the respective impact of ICU-AW and ICU-DD on outcome. Secondly, in these studies, diaphragm dysfunction was defined as Ptr,stim < 11 cmH_2_O, which is the cut-off value currently used in non-ICU patients. However, this cut-off has been recently disputed since a recent report showed that Ptr,stim < 7 cmH_2_O would be the most reliable cut-off to predict weaning failure [10–12], as Ptr,stim < 7 cmH_2_O defines “severe” ICU-acquired diaphragm dysfunction (S-ICU-DD), with weaning failure being the most relevant outcome [13].

We postulated that ICU-DD and ICU-AW have different risk factors with different respective impacts on outcome. To resolve this issue, we merged the population of two pre-existing cohorts [5, 9] and performed a post hoc analysis. The primary objective was to describe the respective risk factors for ICU-AW and S-ICU-DD and the secondary objective was to evaluate the respective impact of ICU-AW and S-ICU-DD on outcome.

## Patients and methods

Detailed methods of the two studies have been previously published [5, 9]. The two studies were conducted in a 10-bed medical ICU in Paris, France (study 1), and in a 16-bed medical surgical ICU in Montpellier, France (study 2), and were approved by the *Comité de Protection des Personnes Ile-de-France V*I (Paris) and the *Comité de Protection des Personnes Sud-Méditerranée* (Montpellier) ethics committees. Informed consent was obtained from all patients or their relatives. Data from these cohorts have been previously published [5, 9, 13, 14].

### Patients

In study 1 [5], patients intubated and ventilated for at least 24 h were eligible for inclusion in the study as soon as they met the predefined readiness-to-wean criteria on daily screening and were therefore deemed ready to undergo a spontaneous breathing trial (SBT). In study 2, patients were eligible for inclusion if they were diagnosed with ICU-AW (defined by a Medical Research Council [MRC] Score < 48), had been mechanically ventilated for at least 48 h, and were undergoing a spontaneous breathing trial.

In both studies, exclusion criteria were contraindications to magnetic stimulation of the phrenic nerves (cardiac pacemaker, implanted defibrillator, or cervical implants), pre-existing neuromuscular disorders (cervical spine injury, bihemispheric or brain stem lesions), and the impossibility to assess limb muscle strength due to immobilization or inability to follow simple instructions.

### Data collection

Demographic data, comorbidities, severity scores, organ dysfunction-related variables, physiological data, blood gas data, medication exposure, duration of MV and ICU stay, and ICU and hospital mortality were prospectively recorded.

#### Diaphragm function

Diaphragm function was assessed in terms of changes in Ptr,stim, [3] in response to bilateral anterior magnetic stimulation of the phrenic nerves [15]. Briefly, two figure-of-eight coils connected to a pair of Magstim® 200 stimulators (The Magstim Company, Dyfed, UK) were positioned immediately posterior to the sternomastoid muscles at the level of the cricoid cartilage. Stimulations were delivered at the maximum intensity allowed by the stimulator. This level of power output is known to produce stimulation which is supramaximal or very close to supramaximal [15].

Patients were studied in a standardized semirecumbent position, as follows: end-expiratory pressure was set to zero, and the patient was allowed to exhale during an end-expiratory pause until expiratory airflow reached zero (relaxed equilibrium volume of the respiratory system). The endotracheal tube was then occluded and bilateral anterolateral magnetic stimulation was performed. Measurements were repeated at least three times by 2 operators to ensure reproducibility. Stimulations were always performed by the same two operators in each center. Ptr,stim was defined as the amplitude of the negative pressure wave following stimulation, measured from baseline to peak at the proximal end of the endotracheal tube, using a linear differential pressure transducer (MP45 ± 100 cmH_2_O, Validyne, Northridge, CA, USA). The pressure signal was sampled and digitized at 128 Hz (MP30, Biopac Systems, Santa Barbara, CA, USA, or Powerlab, AD Instruments, Bella Vista, Australia) for subsequent data analysis.

#### Limb muscle strength

Limb muscle strength was assessed by using the Medical Research Council (MRC) score in patients screened for level of consciousness and understanding.

### Study design

The spontaneous breathing trial was performed after completion of diaphragm and limb muscle assessment. Patients were connected to the ventilator (at pressure support level 7 cmH_2_O with zero end-expiratory pressure, or by T-tube) for a 30-min period. SBT was considered to have failed when criteria of clinical intolerance were present [16]. Otherwise, the spontaneous breathing trial was considered to be successful and patients were extubated when decided by the attending physician. Successful weaning was defined as sustained spontaneous breathing without any form of ventilatory support 48 h after extubation. Weaning failure was defined as patients failing the spontaneous breathing trial or requiring reintubation or any form of ventilatory support (including noninvasive ventilation for post-extubation acute respiratory failure, but not prophylactic noninvasive ventilation) during the 48 h following extubation.

### Definitions

Ptr,stim was used to identify two groups of patients based on the 7 cmH_2_O cut-off already described [13], and patients with Ptr,stim < 7 cmH_2_O were considered to have S-ICU-DD. The MRC score was used to identify two groups of patients based on the cut-off of 48/60 [1], and patients with an MRC score < 48 were considered to have ICU-AW.

### Statistical analysis

Continuous variables are expressed as median (interquartile range), and categorical variables are expressed as absolute and relative frequency. Continuous variables were compared by Kruskal-Wallis or Mann-Whitney *U* test, and categorical variables were compared by the chi-square test or Fisher’s exact test depending on the sample size. We used multiple forward logistic regression models to identify variables independently associated with ICU-AW, S-ICU-DD, weaning failure, and ICU mortality.

To identify the importance of each variable, a classification tree (CART) sequentially partitioned data into homogeneous subsamples. Starting with the complete data set, a partitioning tree searched for the best explanatory variable and the optimal cut-off value (denoted node) in order to obtain two subsamples with increasing purity for class membership. At node 1, two subsamples were obtained with a purity higher than the purity of the initial sample. Each subsample was then partitioned as before, and the best partition was selected. To ensure the robustness of the final model and to avoid potential over-fitting of the data, the model was cross-validated to prune the tree.

For all final comparisons, a *p* value less than or equal to 0.05 was considered to be statistically significant. Analyses were performed using SPSS, v.21 (IBM, Chicago, IL, USA) and R, v. 3.5.0.

## Results

One hundred sixteen patients were enrolled during the study period: 76 in study 1 and 40 in study 2.

### Prevalence and factors associated with ICU-AW and S-ICU-DD

**Fig. 1:**
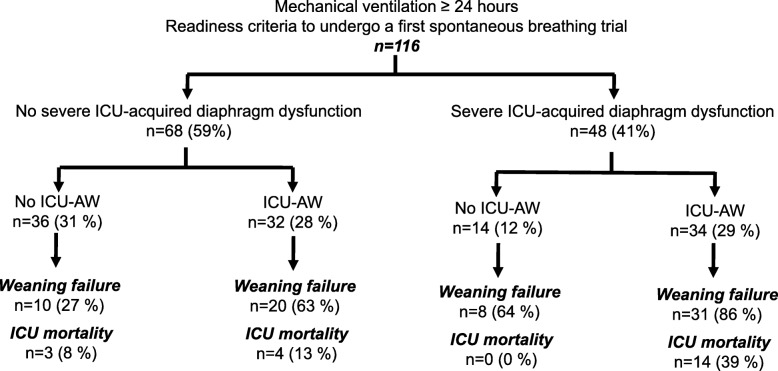
Study flow chart. ICU, intensive care unit; ICU-AW, ICU-acquired weakness

Figure 1 displays the distribution of patients according to the presence of ICU-AW and S-ICU-DD. Sixty-eight (59%) patients had no S-ICU-DD, 36 (31%) of whom had no ICU-AW and 32 (28%) had ICU-AW. Among the remaining 48 patients with S-ICU-DD, 14 (12%) had no ICU-AW and 34 (29%) had ICU-AW.

Table 1 shows patient characteristics according to the presence of ICU-AW and S-ICU-DD. Compared to patients with no S-ICU-DD and no ICU-AW, patients with ICU-AW only had a higher SOFA score on ICU admission and were more frequently exposed to neuromuscular blockers and norepinephrine. Patients with S-ICU-DD only had a higher duration of MV prior to inclusion and were more frequently exposed to corticosteroids. Compared to patients with either S-ICU-DD or ICU-AW, those with both S-ICU-DD and ICU-AW had an even longer duration of MV prior to inclusion and were more frequently exposed to norepinephrine and sufentanil. Multivariate logistic regression analysis identified three factors independently associated with S-ICU-DD: age (odds ratio [OR] 1.08, 95% confidence interval [CI] 1.04–1.13, *p* = 0.0001), exposure to sufentanil (OR 7.38, 95% CI 2.6–23, *p* = 0.0003), and duration of MV prior to inclusion (OR 1.09, 95% CI 1.01–1.19, *p* = 0.028). Two factors were independently associated with ICU-AW: exposure to norepinephrine (OR 7.2, 95% CI 2.9–19, *p* = 0.00003) and duration of MV prior to inclusion (OR 1.27, 95% CI 1.13–1.47, *p* = 0.0003).

**Table 1:** Characteristics of the study population on intensive care unit admission according to the presence of severe intensive care unit-acquired diaphragm dysfunction (S-ICU-DD) and intensive care unit-acquired weakness (ICU-AW)

	No S-ICU-DD		S-ICU-DD		*p*
	No weakness	ICU-AW	No weakness	ICU-AW	
N	36 (31%)	32 (28%)	14 (12%)	34 (29%)	
Demographic data					
Men, *n (%)*	27 (75)	19 (59)	7 (58)	24 (71)	0.522
Age, *years*	58 (40–55)	56 (50–65)	70 (50–78)*	64 (56–75)*^#^	0.009
Body mass index, *kg/m*^*2*^	24 (21–27)	23 (21–26)	24 (20–33)	25 (22–29)	0.302
Medical conditions					
COPD, *n (%)*	7 (19)	0 (0)*	3 (21)^#^	7 (21) ^#^	0.049
Heart failure, *n (%)*	7 (19)	2 (6)	2 (14)	6 (18)	0.453
Reason for ICU admission					
Shock	11 (33)	10 (31)	3 (21)	13 (38)	0.793
Coma	13 (39)	5 (19)	3 (21)	6 (18)	0.186
Acute respiratory failure	12 (33)	17 (53)	8 (57)	15 (44)	0.387
On admission					
SOFA	4 (3–5)	7 (4–10)*	6 (5–8)*	8 (5–13)*	< 0.0001
Sepsis, *n (%)*	24 (67)	23 (72)	7 (58)	23 (68)	0.830
At inclusion					
MV prior to inclusion, *days*	3 (1–5)	4 (4–9)	5 (1–7)*	9 (5–15)*^†^	< 0.0001
Medication exposure					
Neuromuscular blocker, *n (%)*	4 (11)	13 (41)*	4 (28)	17 (50)*	0.017
Corticosteroids, *n (%)*	1 (3)	3 (9)	4 (28)*	1 (3)^†^	< 0.0001
Norepinephrine, *n (%)*	9 (25)	21 (66)*	3 (21)^#^	30 (88)*^†^	< 0.0001
Midazolam, *n (%)*	11 (31)	16 (50)	5 (36)	13 (38)	0.346
Propofol (%)	23 (64)	5 (19)*	7 (50)^#^	5 (15)*^†^	< 0.0001
Sufentanil, *n (%)*	16 (44)	21 (66)	7 (50)	31 (91)* ^†#^	0.001
Muscle assessment					
Ptr,stim, *cmH*_*2*_*O*	10.9 (7.9–15.3)	11.0 (9.0–14.0)	5.0 (4.1–6.3)*^#^	3.5 (2.4–6.3)*^#^	< 0.0001
MRC score	56 (51–59)	35 (23–43)*	55 (51–57)^#^	33 (24–40)*^†^	< 0.0001

Data are expressed as median (interquartile range) or *n* (%)

*COPD* chronic obstructive pulmonary disease, *SOFA* Sequential Organ Failure Assessment, *MV* mechanical ventilation, *Ptr,stim* endotracheal tube pressure induced by bilateral phrenic nerve stimulation during airway occlusion, *MRC* Medical Research Council

*vs. No S-ICU-DD–No ICU-AW

^#^vs. No S-ICU-DD–ICU-AW

^†^vs. S-ICU-DD–No ICU-AW

### Clinical outcomes

Table 2 shows the main clinical outcome. Compared to patients with no S-ICU-DD and no ICU-AW, patients with either S-ICU-DD or ICU-AW presented higher rates of SBT failure and weaning failure. Patients with ICU-AW but no S-ICU-DD had a longer ICU length of stay, in contrast with patients with S-ICU-DD but no ICU-AW. Compared to patients with either S-ICU-DD or ICU-AW, those with both S-ICU-DD and ICU-AW had higher weaning failure and mortality rates.

**Table 2:** Main clinical outcomes according to the presence of severe intensive care unit-acquired diaphragm dysfunction (S-ICU-DD) and intensive care unit-acquired weakness (ICU-AW)

	No S-ICU-DD		S-ICU-DD		*p*
	No weakness	ICU-AW	No weakness	ICU-AW	
*N*	36 (31%)	32 (28%)	14 (12%)	34 (29%)	
Outcomes					
SBT failure, *n (%)*	7 (19)	14 (44)*	7 (58)*	26 (72)*^#^	< 0.0001
Reintubation after extubation following successful SBT, *n (%)*	3 (8)	6 (19)*	1 (8)	8 (23)*	0.039
Weaning failure, *n (%)*	10 (28)	20 (63)*	8 (67)*	31 (86)*^#^	< 0.0001
MV until extubation, *days*	4 (1–6)	7 (4–13)*	7 (3–8)	13 (7–24)*	< 0.0001
ICU length of stay, *days*	5 (3–14)	15 (7–30)*	9 (4–13)^#^	20 (9–30)*^†^	< 0.0001
ICU mortality, *n (%)*	3 (8)	4 (13)	0 (0)	14 (39)*^#†^	0.001

Data are expressed as median (interquartile range) or *n* (%)

*SBT* spontaneous breathing trial, *MV* mechanical ventilation, *ICU* intensive care unit

*vs. No S-ICU-DD–No ICU-AW

^#^vs. No S-ICU-DD–ICU-AW

^†^vs. S-ICU-DD–No ICU-AW

**Fig. 2:**
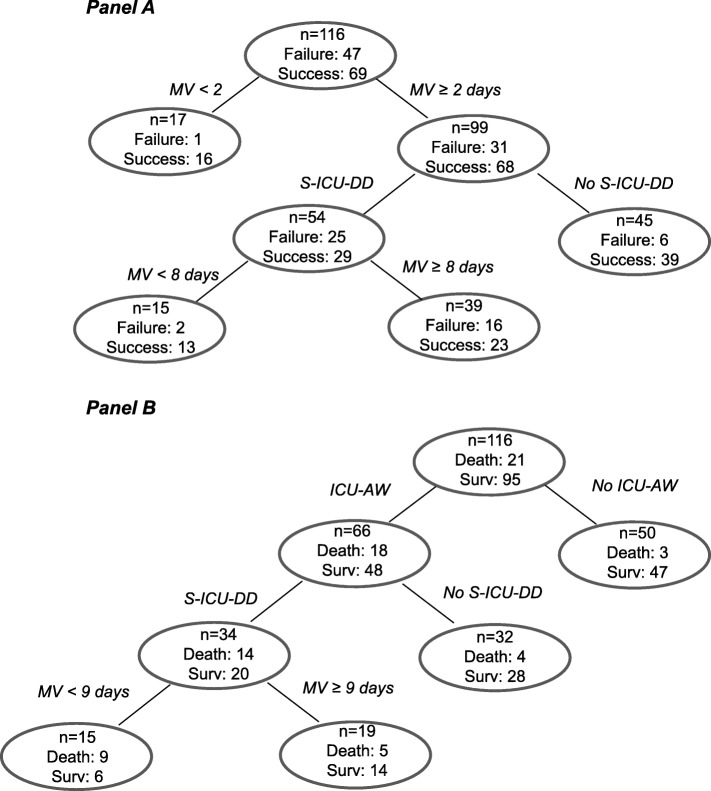
Nonparametric classification and regression tree methodology (CART) selected variables as decision knots, ensuring optimal separation of patients successfully weaned from mechanical ventilation from those not successfully weaned (**a**) and optimal separation of survivors from non-survivors (**b**). MV, duration of mechanical ventilation prior to inclusion; S-ICU-DD, severe intensive care unit-acquired diaphragm dysfunction; ICU-AW, intensive care unit-acquired weakness

The overall weaning failure rate was 59%. Table 3 shows the factors associated with weaning failure identified by univariate analysis. On multivariate logistic regression analysis, two of these factors were independently associated with weaning failure: duration of MV prior to inclusion (OR 1.17, 95% CI 1.04–1.33, *p* = 0.012) and S-ICU-DD (OR 3.56, 95% CI 1.42–9.40, *p* = 0.008). CART analysis confirmed the impact of S-ICU-DD on weaning (Fig. 2, Panel a), as the two variables selected, S-ICU-DD and duration of MV, were most likely to identify patients who were successfully weaned from those with weaning failure.

**Table 3:** Patient characteristics according to weaning outcome

	Weaning failure *n* = 69 (59%)	Weaning success *n* = 47 (41%)	*p*
Men, *n (%)*	44 (75)	33 (70)	0.554
Age, *years*	60 (52–71)	57 (67–41)	0.041
Body mass index, *kg/m*^*2*^	24 (22–28)	24 (22–28)	0.673
Medical conditions			
Chronic heart failure, *n (%)*	9 (13)	8 (17)	0.607
COPD, *n (%)*	13 (19)	4 (9)	0.188
Reason for ICU admission			
Shock, *n (%)*	23 (32)	14 (30)	0.845
Coma, *n (%)*	10 (17)	17 (36)	0.032
Acute respiratory failure, *n (%)*	36 (51)	16 (34)	0.141
On admission			
SOFA	7 (4–11)	5 (4–8)	0.777
Sepsis on admission, *n (%)*	44 (64)	33 (70)	0.473
At inclusion			
MV before inclusion, *days*	7 (4–12)	3 (1–5)	< 0.001
Muscle assessment			
Severe ICU-DD, *n (%)*	57 (83)	22 (47)	< 0.001
Ptr,stim, *cmH*_*2*_*O*	6.7 (3.5–8.4)	11.3 (7.7–15.8)	< 0.001
ICU-AW, *n (%)*	49 (71)	17 (36)	< 0.001
MRC score	36 (30–50)	52 (44–58)	0.014

Data are expressed as median (interquartile range) or *n* (%)

*COPD* chronic obstructive pulmonary disease, *SOFA* Sequential Organ Failure Assessment, *MV* mechanical ventilation, *Ptr,stim* endotracheal tube pressure induced by bilateral phrenic nerve stimulation during airway occlusion, *S-ICU-DD* severe intensive care unit-acquired diaphragm dysfunction, *ICU-AW* intensive care unit-acquired weakness, *MRC* Medical Research Council

Intensive care unit mortality rate was 18%. Table 4 shows the factors associated with mortality identified by univariate analysis. On multivariate logistic regression analysis, two of these factors were independently associated with mortality: ICU-AW (OR 4.30, 95% CI 1.25–20.30, *p* = 0.033) and age (OR 1.04, 95%CI 1.01–1.09, *p* = 0.036). CART analysis confirmed the impact of ICU-AW on mortality (Fig. 2, Panel b).

**Table 4:** Patient characteristics according to outcome

	Survivors *n* = 95 (82%)	Non-survivors *n* = 21 (18%)	*p*
Men, *n (%)*	62 (65)	15 (71)	0.588
Age, *years*	57 (48–67)	65 (56–75)	0.035
Body mass index, *kg/m*^*2*^	24 (21–28)	25 (22–29)	0.282
Medical conditions			
Chronic heart failure, *n (%)*	13 (14)	4 (19)	0.529
COPD, *n (%)*	13 (14)	4 (19)	0.529
Reason for ICU admission			
Shock, *n (%)*	29 (32)	8 (33)	0.876
Coma, *n (%)*	25 (28)	2 (14)	0.181
Acute respiratory failure, *n (%)*	41 (43)	11 (52)	0.442
On admission			
SOFA	5 (4–9)	8 (4–15)	0.100
Sepsis admission	61 (64)	16 (76)	0.293
At inclusion			
MV before inclusion, *days*	5 (2–9)	7 (5–12)	0.056
Muscle assessment			
S-ICU-DD, *n (%)*	34 (36)	14 (67)	0.009
Ptr,stim, *cmH*_*2*_*O*	8.4 (5.7–13.0)	4.1 (2.8–8.4)	0.001
ICU-AW, *n (%)*	48 (50)	18 (86)	0.005
MRC score	47 (35–55)	32 (19–51)	< 0.0001

Data are expressed as median (interquartile range) or *n* (%)

*COPD* chronic obstructive pulmonary disease, *SOFA* Sequential Organ Failure Assessment, *MV* mechanical ventilation, *Ptr,stim* endotracheal tube pressure induced by bilateral phrenic nerve stimulation during airway occlusion, *S-ICU-DD* severe intensive care unit-acquired diaphragm dysfunction, *ICU-AW* intensive care unit-acquired weakness, *MRC* Medical Research Council

## Discussion

The main results of our study are as follows: (1) some of the risk factors associated with S-ICU-DD and S-ICU-AW are different; (2) S-ICU-DD, but not ICU-AW, is independently associated with weaning failure, while ICU-AW, but not S-ICU-DD, is associated with ICU mortality; and (3) the impact of the combination of S-ICU-DD and ICU-AW on outcome is more pronounced than the individual impact of each entity.

We observed that some of the risk factors associated with S-ICU-DD and ICU-AW were different, which is consistent with core physiological data suggesting that ICU-DD and ICU-AW may be associated with distinct risk factors [17–21]. Here, exposure to sufentanil was associated with S-ICU-DD but not ICU-AW, which reminds us that the use of opioids should be limited in quantity in ICU patients [22]. Clearly, diaphragm and limb muscles are different, and they are not equally vulnerable to a given injury. This is for instance the case of pneumonia, sepsis, or MV, to which the diaphragm is more vulnerable [1, 18, 23–28]. Conversely, limb muscles are more vulnerable to hemorrhagic shock [29]. This unequal vulnerability might be explained by differences in terms of activity [30], microcirculation [29], cytokines [23], chemokines [23], and free radicals involved in oxidative stress [31]. However, the purely observational design of this study precludes any conclusions regarding why and how a given factor associated with ICU-acquired muscle weakness more specifically targets the diaphragm or limb muscles. Further studies are clearly needed. It is noteworthy that, although the two entities appeared to be partially independent, a substantial number of patients presented both S-ICU-DD and ICU-AW. These patients presented a combination of risk factors associated with S-ICU-DD and risk factors associated with ICU-AW.

In the present study, as previously observed, ICU-AW and S-ICU-DD each had an individual impact on outcome [1, 5, 8]. However, we showed that these two entities had a different individual impact on outcome, as S-ICU-DD but not ICU-AW was independently associated with weaning failure, and ICU-AW but not S-ICU-DD was associated with ICU mortality. This finding highlights the fact that diaphragm and limb muscles may have different roles in critically ill patients. The diaphragm is the main inspiratory muscle, which is why the diaphragm pump is essential for spontaneous breathing, at least in the ICU where most patients experience increased respiratory loading. Patients with severe diaphragm dysfunction are subsequently at high risk of spontaneous breathing trial failure [5, 13]. A recent study showed that diaphragm dysfunction is associated with prolonged weaning [32]. Following extubation, patients with severe diaphragm dysfunction may also be unable to sustain spontaneous breathing [33]. Low tidal volume due to diaphragm dysfunction is likely to cause atelectasis [34], and these patients are at high risk of immediate post-extubation acute respiratory failure [5].

With a few exceptions, weaning from MV is a mandatory, but not sufficient, condition to remain alive. However, extubated patients must also be able to drain and evacuate even copious secretions, which is why adequate cough and the ability to swallow are important extubation criteria. Weak cough is definitely a major cause of delayed post-extubation acute respiratory failure [35] that may ultimately increase mortality [36]. In addition, ICU-AW induces bed rest, which, in turn, promotes atelectasis, hospital-acquired infections including pneumonia, thrombophlebitis, and skin damage [37, 38]. ICU-AW is associated with weak cough, which may explain why ICU-AW is independently associated with mortality, while S-ICU-DD is not. In addition, we observed that patients with both S-ICU-DD and ICU-AW had a poorer outcome than patients with either ICU-AW or S-ICU-DD. To the best of our knowledge, this study shows for the first time that S-ICU-DD and ICU-AW have a cumulative impact on outcome.

The strengths of our study include the largest cohort of critically ill patients in whom diaphragm function and limb muscle strength have been evaluated, the fact that our cohort included patients from two centers and the use of Ptr,stim to study diaphragm function. In addition, we focused on severe ICU-DD rather than ICU-DD because severe ICU-DD is more reliable to predict weaning failure than ICU-DD [13].

Our study presents several limitations. Firstly, the use of phrenic nerve stimulation as the reference technique to define diaphragm dysfunction excluded some eligible patients in whom this technique was contraindicated [39]. Secondly, ICU-AW was diagnosed by means of the MRC score after ruling out causes of limb weakness other than critical illness. Although the accuracy of the MRC score has been questioned [40], it is reproducible [41] and performed routinely in the two participating ICUs. Dynamometry or measurement of adductor pollicis muscle function by magnetic stimulation of the ulnar nerve [42] is not as reproducible as MRC score and is much more time-consuming. Thirdly, the patients were eligible for inclusion if they were intubated and ventilated for at least 24 h in study 1 and 48 h in study 2. Although it may create a selection bias, we feel that it is not major. In addition, it creates a little more heterogeneous population, which is closer to what is encountered in daily practice.

## Conclusion

In conclusion, in a prospective cohort of 116 critically ill MV patients, the risk factors associated with ICU-AW and S-ICU-DD were different, highlighting the fact that these two entities may be due to distinct mechanisms. These mechanisms and the reason why they differ between the diaphragm and limb muscles need to be more clearly elucidated. S-ICU-DD and ICU-AW also have different impacts on mortality, as ICU-DD is independently associated with weaning failure and ICU-AW is independently associated with mortality. In addition, the impact of the combination of ICU-DD and ICU-AW on outcome is more pronounced than their individual impact. These results highlight the fact that ICU-DD and ICU-AW should be evaluated separately in critically ill MV patients.

## Data Availability

The datasets used and/or analyzed during the current study are available from the corresponding author on reasonable request.

## Abbreviations

ICU: Intensive care unit
ICU-AW: Intensive care unit-acquired weakness
S-ICU-DD: Severe intensive care unit-acquired diaphragm dysfunction
MV: Mechanical ventilation
CART: Classification and regression *tree*
SBT: Spontaneous breathing trial
MRC: Medical Research Council
